# Temperature Dependence of the Pore Structure in Polyvinylidene Fluoride (PVDF)/Graphene Composite Membrane Probed by Electrochemical Impedance Spectroscopy

**DOI:** 10.3390/polym10101123

**Published:** 2018-10-10

**Authors:** Qizhao Luo, Qing Huang, Zhe Chen, Lei Yao, Qiuming Fu, Ping Fu, Zhidong Lin

**Affiliations:** 1 Hubei Key Laboratory of Plasma Chemical and Advanced Materials & School of Materials Science and Engineering, Wuhan Institute of Technology, Wuhan 430205, China; MagicianTosaka@163.com (Q.L.); a635442574@163.com (Q.H.); qiumingfuwit@163.com (Q.F.); fuping751128@163.com (P.F.); Zhidong.lin@126.com (Z.L.); 2 School of Electrical and Information Engineering, Wuhan Institute of Technology, Wuhan 430205, China

**Keywords:** pore structure, PVDF/graphene membrane, temperature dependence, electrochemical impedance spectroscopy

## Abstract

In this paper, graphene was introduced in the PVDF to improve the thermal stability of the pore structure, which is the key feature for the membrane applied for the thermo-osmotic energy conversion (TOEC) process. The PVDF/graphene composite membranes were characterized by a scanning electron microscopy (SEM), a water contact angle measurement, and electrochemical impedance spectroscopy (EIS). It was found that the composite membranes exhibited improved surface hydrophobicity. Moreover, the pores in pure PVDF membrane would expand during the heat process while the existence of graphene in PVDF clearly suppressed the expansion, which implied better thermal stability of the pores in the composite membrane. According to the pore deformation time, the heat conductivities of the membranes were calculated and compared with each other. It confirmed that the composite membrane with higher graphene content exhibited enhanced heat conductivity. EIS can be used to monitor the temperature dependence of the pore structure in aqueous environments.

## 1. Introduction

There is a vast amount of unused energy in the form of low-grade heat (below 100 °C) in the world [1]. However, it is difficult to extract energy from the low-grade heat through traditional technologies due to the small temperature difference available from the waste heat sources. Recently, thermo-osmotic energy conversion (TOEC) was introduced as an approach for generating energy from low-grade heat [2,3,4]. Due to the temperature difference, thermo-osmotic liquid was driven through the membrane against a hydraulic pressure difference, which can be de-pressurized through a turbine to generate electricity. Elimelech and colleagues used a hydrophobic porous membrane in the TOEC process and found the pore structure of the membrane was essential for the system efficiency [2]. Since the temporal variability in heat output from the sources such as waste heat or solar thermal power cannot be controlled, membranes with thermally stable pore structures were preferred.

Polyvinylidene fluoride (PVDF) as a semi-crystalline polymer exhibits high mechanical strength, thermal stability, and excellent aging resistance [5], which are important for the application of the TOEC process. Graphene is a monolayer of carbon atoms, which has been successfully used as an additive in polymers in order to reinforce the thermal conductivity and mechanical strength. The incorporation of graphene can also enhance the hydrophobicity of the polymer due to its intrinsic hydrophobic nature [6]. Therefore, the PVDF/graphene membrane can be a candidate for the application in the TOEC process. In order to obtain the optimized membrane for the TOEC process, it is necessary to know the temperature dependence of the pore structures of the membrane.

Various characterization techniques have been successfully applied to investigate the membrane structures such as SEM [7,8], TEM [9,10], porometric detection [11,12], AFM [13,14], and positron annihilation [15,16]. The information about pore structures can be directly or indirectly obtained through the above techniques. However, it is difficult for these methods to probe the temperature dependence of the pore structure of the membrane in an aqueous environment.

Electrochemical impedance spectroscopy (EIS) is a nondestructive technique, which is based on the electrochemical relaxations related to the intrinsic properties of the sample. Since the electrical properties are related to the membrane states such as porosity and surface charges, EIS can be applied as an in situ real-time method to study the membrane structures [17,18,19]. The change in resistance and capacitance in EIS data directly reflects the difference in membrane states. Moreover, during the EIS measurement, the membrane needs to be immersed in the electrolyte, which is similar to the TOEC process.

Therefore, EIS was applied to probe the temperature dependence of the pore structures of the PVDF/graphene membranes in this study. The effect of the graphene on the thermal stability of the pore structure in a composite membrane was discussed.

## 2. Materials and Methods

### 2.1. Materials

Polyvinylidene fluoride (PVDF, TA-6010) powders were purchased from Solvay Company (Brussels, Belgium). Graphene was supplied by Tanfeng Tech, Inc. (Suzhou, China). *N*,*N*-Dimethylformamide (DMF), polyethylene glycol (PEG, *M*_w_ = 1000) and KCl were purchased from the Sinopharm Chemical Reagent, Co., Ltd. (Beijing, China). All the chemicals were used as received.

### 2.2. Preparation of PVDF/graphene Membranes

PVDF/graphene solutions were prepared by dissolving the PVDF (2 g), graphene (the weight of graphene was 2, 10, 20, and 30 mg, respectively), and PEG (80 mg) in DMF (10 mL). The sample was labeled as PVDF/0.1%, PVDF/0.5%, PVDF/1.0%, and PVDF/1.5% graphene, respectively.

The mixture was kept 60 °C for 6 h to obtain a homogenous solution by using a magnetic stirrer and then stood still for 12 h for degassing. Consequently, the solutions were cast by a 400-μm knife on a flat glass. These obtained membranes were immersed into deionized water for 24 h. Lastly, the membranes were placed in the oven and kept for 60 °C for 6 h.

### 2.3. Characterizations

The cross-section morphologies of the membranes were observed by using a scanning electron microscopy (SEM Hitachi SU8020, Hitachi, Tokyo, Japan). The membranes were first immersed in the liquid nitrogen for 1 min and then broken. Consequently, the obtained cross-sections were coated with Au for SEM characterization.

The hydrophobic state of the membrane surface was evaluated by a contact angle measuring device (SL2006) from Shanghai Kino Tech Corp (Shanghai, China). The water contact angles were calculated by a circle fitting to a distilled water drop of 1 μL. For each sample, the contact angle measurements were repeated at five different sites.

EIS measurements were performed by a CS310H impedance analyzer operating in a frequency range from 1 MHz to 0.1 Hz with a traditional double electrode. The membrane was fixed between two electrolytic cells and the effective area of the testing membrane was 7 cm^2^. The electrolyte solution was heated to a designated temperature and then injected in the electrolytic cell while the water bath was used to maintain the temperature for the system. Two platinum electrodes with an area of 1 cm^2^ were installed in the two electrolytic cells to maintain the same interval and depth of immersion. The apparatus for EIS measurements at different temperatures were shown in Figure S1.

## 3. Results and Discussion

### 3.1. Membrane Morphology

**Figure 1:**
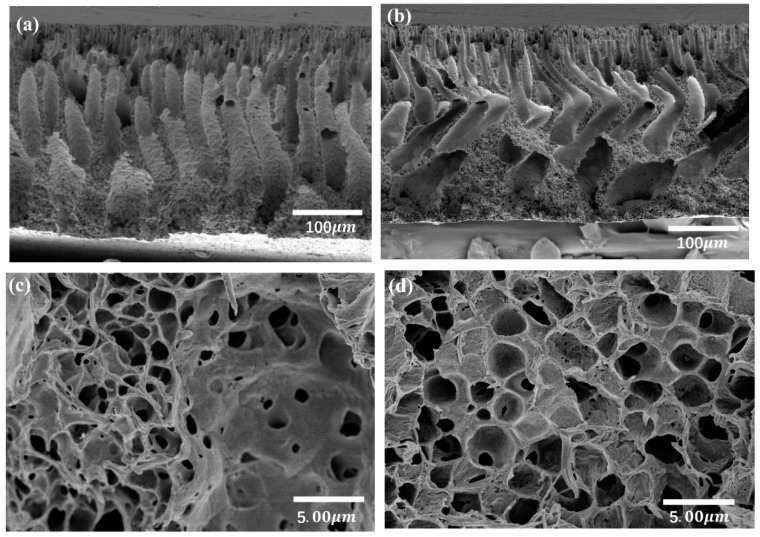
SEM cross-section of the PVDF membrane as prepared samples (**a**,**c**) as well as after the samples were immersed in hot water (**b**,**d**).

**Figure 2:**
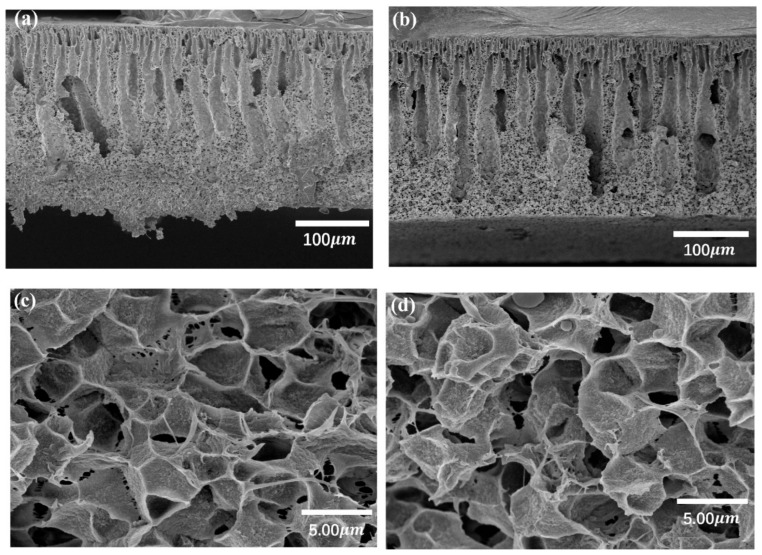
SEM cross-section of the PVDF/1% graphene membrane as prepared samples (**a**,**c**) as well as the samples after being immersed in hot water (**b**,**d**).

The cross-section morphologies of the pure PVDF and PVDF/graphene membrane were shown in Figure 1 and Figure 2, respectively. For the pure PVDF membrane, compared with the as prepared one, the one immersing in deionized water for 1 h at 50 °C exhibits expanded pores. The finger-like pores exhibit distorted shapes after being immersed in hot water. This may contribute to the rearrangement of the polymer chains at a higher temperature [20,21]. For the PVDF/graphene membrane, there is no obvious change in the cross-section morphologies after being immersed in the water for 1 h at 50 °C. The existence of graphene sheets in the matrix can enhance the thermal stability of the composites, which has been confirmed by many research studies [22,23]. Thus, the thermal stability of the pore structure in the composite membrane should be attributed to the graphene introduced in the membrane. Lastly, it was found that the morphology of the PVDF/graphene membrane is rougher than the pure PVDF membrane, which is similar to other results [24,25]. Since the graphene is an extreme hydrophobic material [26,27], the water molecules prefer to contact the area without graphene during the phase conversion stage. In this case, the cross-section morphology in the composite membrane seems to be rougher than the pure membrane.

### 3.2. Surface Hydrophobicity of the Membrane

**Figure 3:**
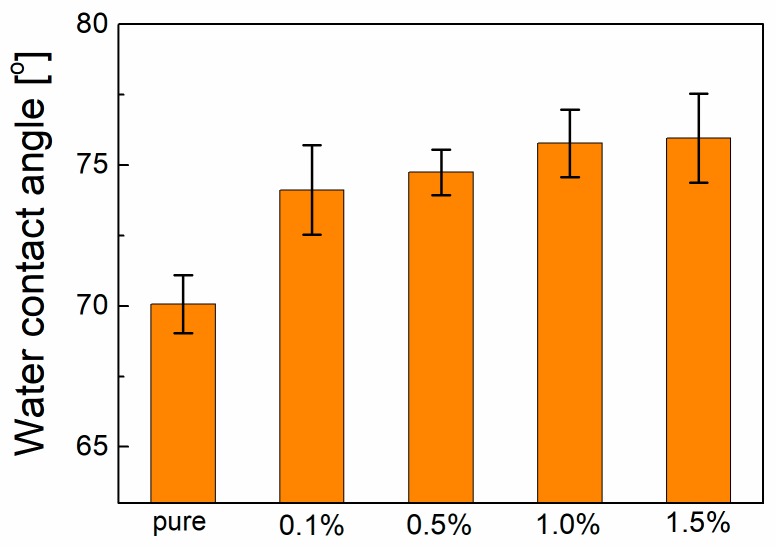
Water contact angle of the membranes.

The hydrophobic membrane is suitable for the TOEC process. Thus, the surface hydrophobicity of the membrane was evaluated by the water contact angle measurement. These results are shown in Figure 3. It was clearly found that the contact angle increased with the addition of the graphene. This should be due to the significant hydrophobicity of the graphene. Introducing the graphene in the PVDF endows a more hydrophobic surface than the pure PVDF membrane. The similar results have been observed in polymer/graphene composites [6]. Therefore, the incorporation of graphene in PVDF can improve the surface hydrophobicity, which is helpful for the TOEC process.

### 3.3. The Temperature Dependence of the Pore Structure

**Figure 4:**
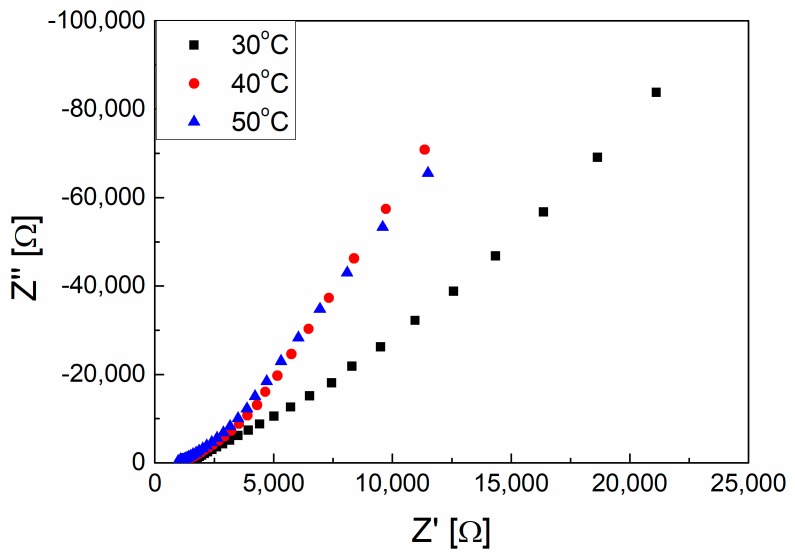
The Nyquist plots of 0.1 mol/L KCl solution at 30, 40, and 50 °C, respectively.

The pore structure of the membrane is first evaluated by EIS in this part. In order to eliminate the influence from the system, (such as the electrolytes/electrode double layer, solution resistance, etc.), the empty system was detected by EIS at a different temperature. The Nyquist plots were shown in Figure 4 and the fitting results were shown in Table 1. Apparently, the solution resistance decreases with a rising temperature and this not only contributed to the change of dielectric constant but also due to the elimination of the concentration polarization by heating.

**Figure 5:**
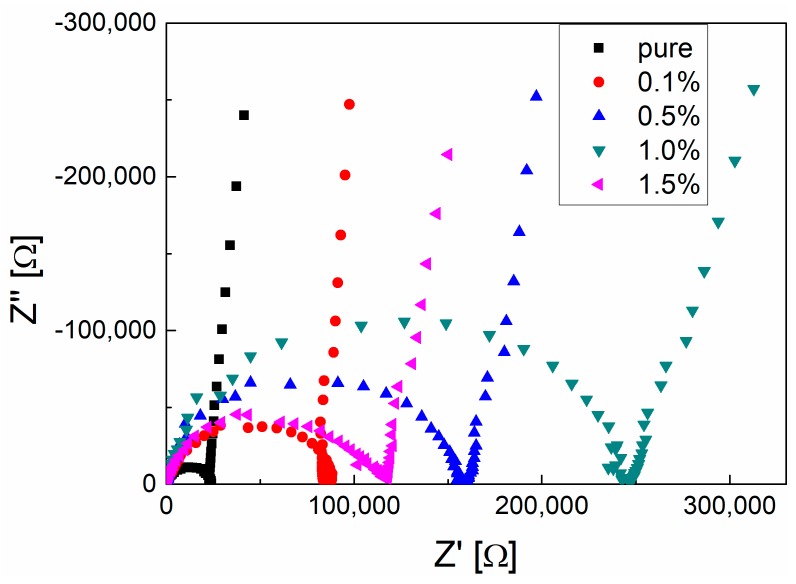
The Nyquist plot of the PVDF membrane with different graphene contents immersing in 0.1 mol/L KCl solution at room temperature.

The Nyquist plots of membranes with different graphene contents in 0.1 mol/L KCl aqueous solution are shown in Figure 5. Compared to the Nyquist plot of the electrolyte solution, the apparent semicircle in Figure 5 is caused by the membrane. It can be clearly found that the semicircle becomes larger with an increase in graphene content from 0.1% to 1.0% and then becomes smaller with graphene content increasing to 1.5%.

**Figure 6:**
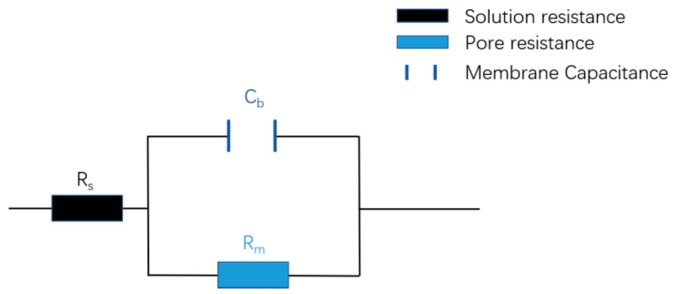
The equivalent circuit model for the present system.

According to our previous studies [28,29], the present system can be analyzed by an equivalent circuit model, which is shown in Figure 6. In this case, the real and imaginary parts of EIS results can be expressed as Equations (1)–(3) in the following.

(1) $$Z_{r}=R_{s}+\frac{R_{m}}{1+{\left(\omega R_{m}C_{b}\right)}^{2}}$$

(2) $$Z_{j}=\frac{\omega C_{b}{R_{m}}^{2}}{1+{\left(\omega R_{m}C_{b}\right)}^{2}}$$

(3) ω=2πf

where *R*_s_ and *R*_m_ correspond to the solution resistance and membrane resistance, respectively. *C*_b_ is the membrane capacitance and *ω* is the angular frequency. The scanning frequency *f* of our apparatus is from 1 MHz to 0.1 Hz, which starts with the minimum point to the maximum point in the abscissa.

The membrane resistances can be obtained from the Nyquist plots by using the above equivalent circuit and the results are presented in Table 2.

According to the pore-impedance relationship, the membrane resistance *R*_m_ can be attributed to the impediment to the ions passing through the pores [30,31], which can be computed by the Equation below [32].

(4) $$R_{m}=\rho \frac{\tau l}{\varepsilon \pi r_{p}^{2}}$$

where *ρ* is the electrical resistivity of electrolyte, *ε* is the volumetric porosity, *r*_p_ is the pore radius, *τ* is the tortuosity, and *l* is the thickness of the membrane. For the polymer/graphene system, introducing graphene into the polymer matrix would result in more tortuous routes for passing ions [28], which leads to an increase of the tortuosity *τ*. As a result, the membrane resistance increases when increasing the graphene content from 0% to 1.0%. Many studies have found that introducing graphene with a higher amount would lead to the aggregation of those nano-sheets [33,34]. Thus, the decline of the membrane resistance for the sample with the graphene content of 1.0% may be due to the aggregation of graphene platelets in the matrix. Therefore, introducing graphene in the PVDF membrane would weaken the permeability of the membrane.

**Figure 7:**
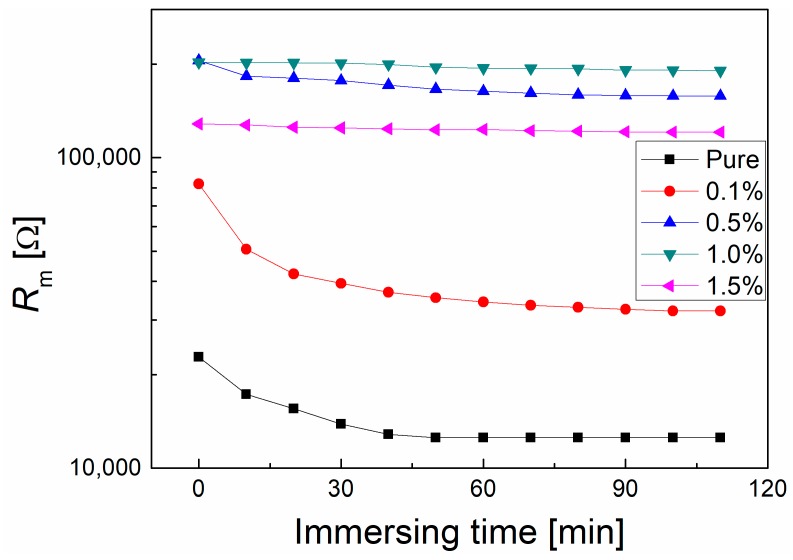
The plots of membrane resistance vs. immersing time for different PVDF membranes immersing 0.1 mol/L KCl solution at 30 °C.

The most important feature for the membrane applied in the TOEC process is the thermal stability of the pore structure since the output energy is influenced by the pore structure. Thus, the temperature dependences of the pore structures of the membranes were evaluated by EIS. The plots of membrane resistance vs. immersing time for all the membranes in KCl solution at 30 °C are shown in Figure 7. This indicated that the pure PVDF membrane experienced a decrease of membrane resistance during the first 40 min while, for the membranes with graphene content higher than 0.5%, the membrane resistances were nearly unchanged during the immersing time. This implies that the thermal stability of the pore structure in the composite membrane is better than the pure one at 30 °C.

**Figure 8:**
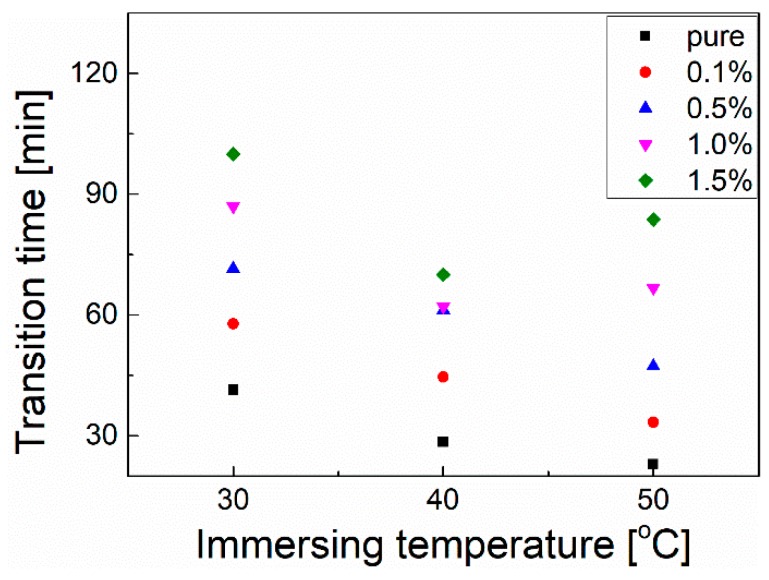
The transition time vs. the immersing temperature for the membranes immersed in the solution.

To further investigate the temperature dependence of the pore structures for each membrane, the plots of the resistance vs. immersing time at different temperatures are shown in Figure S2. It indicates that higher immersing temperature would lead to a lower membrane resistance, which implies more tunnels in the membrane for ion permeation corresponding to a more porous structure of the membrane [32]. It is interesting to find that all the plots first exhibit a decline more or less and then keep a constant value. Thus, we plot the temperature and time of the transition point and the results are presented in Figure 8. It indicates that more graphene content can result in a longer time for transition, which suggests that enhanced thermal stability of the pore structure is due to the existence of graphene.

The pore expanding during the immersing process should be attributed to the chain segments relaxation and this relaxation would be determined by temperature [20,21]. As a result, the thermal stability of the pores is related to the heat conduction of the composite membranes during the immersing process. Thus, it is necessary to compare the heat conductivities of the membranes. According to the definition of heat conductivity [35], the conducted energy through the membrane can be calculated by the Equation below.

(5) $$Q/t=\lambda \left(T_{2}-T_{1}\right)/l$$

where *Q* is the heat energy through the membrane, *t* is the time, *λ* is the heat conductivity, *l* is the thickness of the membrane, and *T*_1_ and *T*_2_ are the original temperatures outside and inside the membrane.

Assuming that the heat energy inducing pore expansion is the same for all the membranes, the ratio of the heat conductivity of the composite membrane to that of the pure PVDF can be obtained by using the Equation below.

(6) $$\frac{\lambda_{\mathrm{composite}}}{\lambda_{\mathrm{pure}}}=\frac{t_{\mathrm{pure}}}{t_{\mathrm{composite}}}*\frac{T_{2p}-T_{1p}}{T_{2c}-T_{1c}}$$

**Figure 9:**
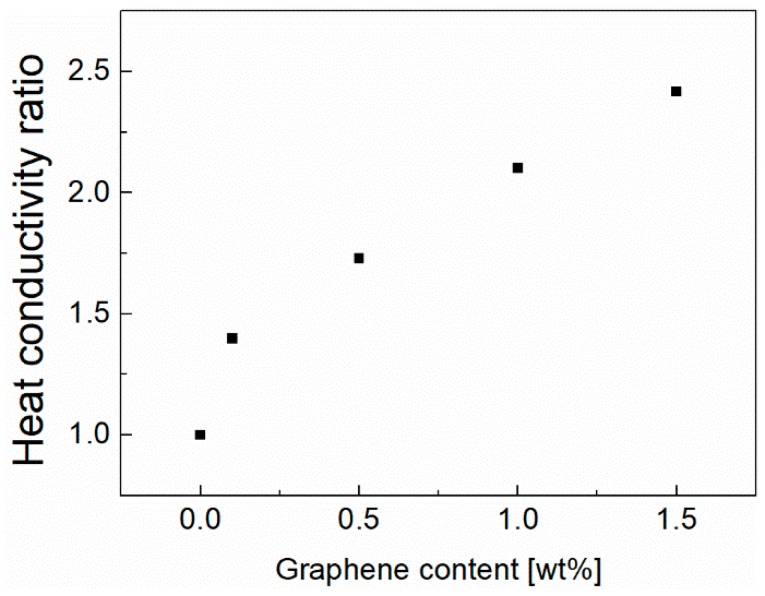
The variation of the heat conductivity ratio of the membranes as a function of the graphene content.

The calculated results are shown in Figure 9. It can be clearly shown that the heat conductivity increases with increasing graphene content. Since the graphene exhibits perfect heat conduction due to the strong covalent sp2 bonding [36], introducing the graphene in the PVDF membrane can improve the heat conduction. On the other hand, the high specific surface area of graphene enables it to fully integrate and interact with the PVDF matrix, which improves the thermal conductivity of the composite materials. Higher graphene content would favor the heat conduction of the composite membrane, which should be contributed to the formation of the heat conduction path. This path was promoted by the graphene sheets that were in contact with each other.

Therefore, the improved thermal stability of the pores in composite membranes can be attributed to two factors as the following. First, the dispersion of graphene in the PVDF membrane can improve the heat conduction and avoid the local overheating during the immersion process. Second, graphene can significantly improve the mechanical properties of the composite membrane, which results in the difficulty for the migration of the chain segments [37]. As a result, the thermal stability of the pore structure is improved by the existence of graphene, which may be a clue for developing novel membranes for the TOEC process.

## 4. Conclusions

The temperature dependence of the pore structures of PVDF/graphene membranes were evaluated in the present study. It was found that the pores would expand when immersing the PVDF membrane in hot aqueous solution while introducing graphene sheets into the PVDF membrane can suppress the expansion, which can be explained by the improved heat conductivity of the composite membrane. The PVDF/graphene composite membrane exhibits improved surface hydrophobility. All the results suggest the PVDF/graphene composite membrane can be applied as a candidate membrane for TOEC. EIS can be utilized as a simple real-time non-destructive method to monitor the pore structure during the TOEC processes in the aqueous environment.

## Figures and Tables

**Table 1:** The electrolyte solution resistance *R*_s_ from Figure 4.

Temperature (K)	*R*_s_ [Ω]
303	1480
313	1140
323	990

**Table 2:** The fitted membrane resistance *R*_m_ from the Nyquist plot, according to the equivalent circuit.

Membrane	*R*_m_ [Ω]
Pure PVDF	22,796
PVDF/0.1% graphene	82,259
PVDF/0.5% graphene	205,290
PVDF/1.0% graphene	245,000
PVDF/1.5% graphene	128,540

## Supplementary Materials

The following are available online at http://www.mdpi.com/2073-4360/10/10/1123/s1, Figure S1: The schematic of the EIS measurement setup. Both of the electrolytic cell and the water bath were placed in the Faraday cage, Figure S2: The variation of membrane resistance as a function of the immersing time for the membranes at 30, 40 and 50 °C, respectively.
