# Supplementary material for: Temperature Dependence of the Pore Structure in Polyvinylidene Fluoride (PVDF)/Graphene Composite Membrane Probed by Electrochemical Impedance Spectroscopy
Source: Polymers (Basel). 2018 Oct 10;10(10):1123. doi: 10.3390/polym10101123 (PMC6403548; doi:10.3390/polym10101123)
Supplement: Supplementary file 1 [file polymers-10-01123-s001.pdf]

# Temperature Dependence of the Pore Structure in Polyvinylidene Fluoride (PVDF)/ Graphene Composite Membrane Probed by Electrochemical Impedance Spectroscopy

**Qizhao Luo[1], Qing Huang[1], Zhe Chen[1,*], Lei Yao[2*], Qiuming Fu[1], Ping Fu[1] and Zhidong Lin[1]**

[1] Hubei Key Laboratory of Plasma Chemical and Advanced Materials & School of Materials Science and Engineering, Wuhan Institute of Technology, Wuhan, 430205, China
[2] School of Electrical and Information Engineering, Wuhan Institute of Technology, Wuhan, 430205, China
* Correspondence: zhezchen@gmail.com; LYAO1@e.ntu.edu.sg Tel: 86-27-87195661

### S1.The EIS measurement setup

The apparatus for EIS measurements at different temperatures were shown in Figure S1.

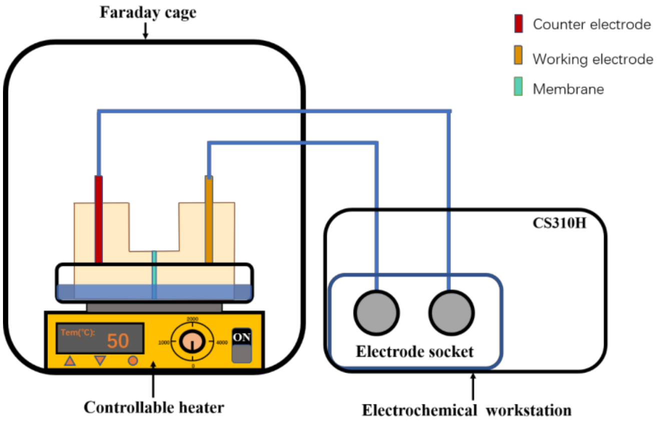


**Figure S1.**The schematic of the EIS measurement setup. Both of the electrolytic cell and the water bath were placed in the Faraday cage.

### S2.The EIS results for membrane immersing in solution at different temperature

To further investigate the temperature dependence of the pore structures for each membrane, the plots of the resistance vs. immersing time at different temperatures are shown in Fig. S2. It indicates that higher immersing temperature would lead to a lower membrane resistance, which implies more tunnels in the membrane for ions permeation, corresponding to more porous structure of the membrane. Moreover, compared with the pure PVDF membrane, the composite membranes exhibit a more gradual decline of the membrane resistance with increasing immersing time, which suggests that introducing graphene in the membrane can

endow thermal stability to the pore structures of the composite membranes. Besides, it is interesting to find that all the plots firstly exhibit a decline more or less and then keep a constant value.

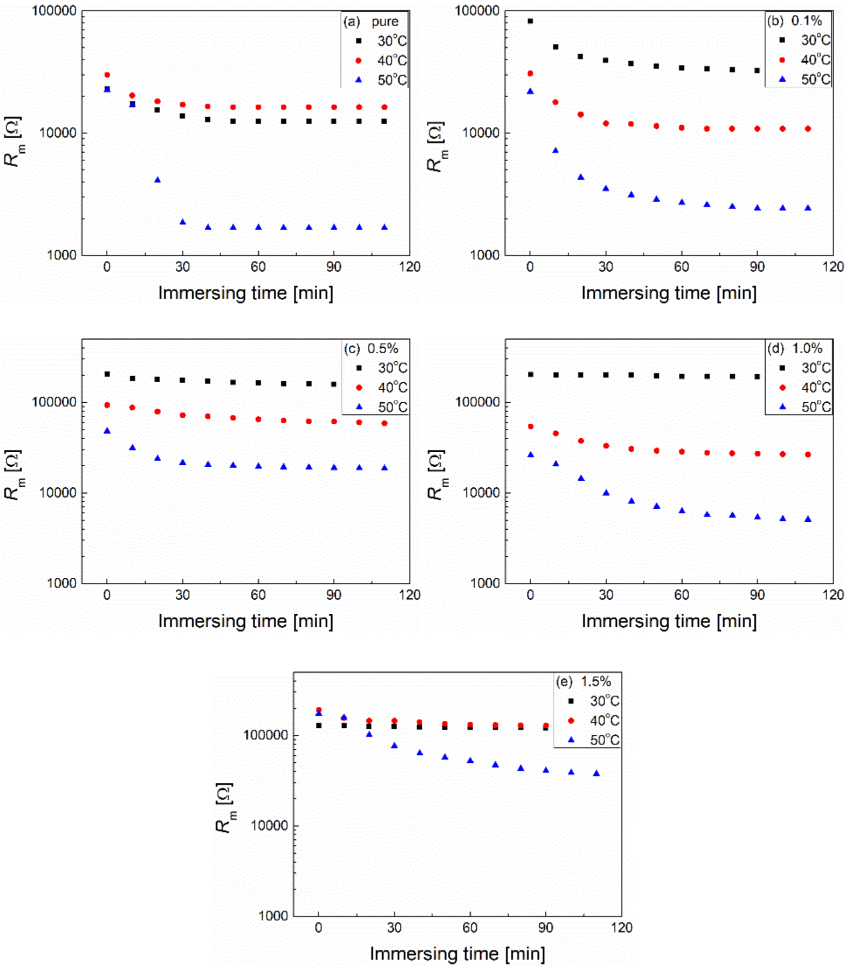


**Figure S2.**The variation of membrane resistance as a function of the immersing time for the membranes at 30, 40 and 50 °C, respectively.
